# Energy and protein requirements for children with CKD stages 2-5 and on dialysis–clinical practice recommendations from the Pediatric Renal Nutrition Taskforce

**DOI:** 10.1007/s00467-019-04426-0

**Published:** 2019-12-16

**Authors:** Vanessa Shaw, Nonnie Polderman, José Renken-Terhaerdt, Fabio Paglialonga, Michiel Oosterveld, Jetta Tuokkola, Caroline Anderson, An Desloovere, Laurence Greenbaum, Dieter Haffner, Christina Nelms, Leila Qizalbash, Johan Vande Walle, Bradley Warady, Rukshana Shroff, Lesley Rees

**Affiliations:** 1 University of Plymouth, Plymouth, PL6 8BH UK; 2 University College London Institute of Child Health, London, UK; 3 British Columbia Children’s Hospital, Vancouver, Canada; 4 Wilhelmina Children’s Hospital, University Medical Center Utrecht, Utrecht, The Netherlands; 5 Fondazione IRCCS Ca’Granda Ospedale Maggiore Policlinico, Milan, Italy; 6 Emma Children’s Hospital, Amsterdam University Medical Center, Amsterdam, The Netherlands; 7 Children’s Hospital and Clinical Nutrition Unit, Internal Medicine and Rehabilitation, University of Helsinki and Helsinki University Hospital, Helsinki, Finland; 8 Southampton Children’s Hospital, University Hospital Southampton NHS Foundation Trust, Southampton, UK; 9 University Hospital Ghent, Ghent, Belgium; 10 Emory University and Children’s Healthcare of Atlanta, Atlanta, USA; 11 Children’s Hospital, Hannover Medical School, Hannover, Germany; 12 PedsFeeds LLC, University of Nebraska, Lincoln, USA; 13 Great Northern Children’s Hospital, Newcastle Upon Tyne, UK; 14 Children’s Mercy, Kansas City, USA; 15 Great Ormond Street Hospital for Children NHS Foundation Trust, London, UK; 16 The Biomedical Research Centre at Great Ormond Street Hospital for Children NHS Foundation Trust and Institute of Child Health, University College London, London, UK

**Keywords:** Energy, Protein, Chronic kidney disease, Pediatric Renal Nutrition Taskforce, Clinical practice recommendations

## Abstract

Dietary management in pediatric chronic kidney disease (CKD) is an area fraught with uncertainties and wide variations in practice. Even in tertiary pediatric nephrology centers, expert dietetic input is often lacking. The Pediatric Renal Nutrition Taskforce (PRNT), an international team of pediatric renal dietitians and pediatric nephrologists, was established to develop clinical practice recommendations (CPRs) to address these challenges and to serve as a resource for nutritional care. We present CPRs for energy and protein requirements for children with CKD stages 2–5 and those on dialysis (CKD2–5D). We address energy requirements in the context of poor growth, obesity, and different levels of physical activity, together with the additional protein needs to compensate for dialysate losses. We describe how to achieve the dietary prescription for energy and protein using breastmilk, formulas, food, and dietary supplements, which can be incorporated into everyday practice. Statements with a low grade of evidence, or based on opinion, must be considered and adapted for the individual patient by the treating physician and dietitian according to their clinical judgment. Research recommendations have been suggested. The CPRs will be regularly audited and updated by the PRNT.

**Electronic supplementary material:**

The online version of this article (10.1007/s00467-019-04426-0) contains supplementary material, which is available to authorized users.

## Introduction

Poor nutrition is one of the best described causes of poor growth in children with chronic kidney disease (CKD) (1). Malnutrition is also associated with worsening uremic symptoms and can lead to protein-energy wasting and increased mortality. Conversely, obesity is a worldwide problem that is also increasingly affecting the CKD population (2).

In 2009, the National Kidney Foundation Kidney Disease Outcomes Quality Initiative (KDOQI) published comprehensive nutritional guidelines (3). The Pediatric Renal Nutrition Taskforce (PRNT) provides clinical practice recommendations (CPRs), updated wherever there is new evidence subsequent to the publication by KDOQI, on various aspects of the dietary management of children with kidney diseases; this document focuses on energy and protein requirements. The composition of the PRNT, limitations, and grading of evidence, and plans for audit and revision of the CPRs have been previously described (4).

Nutritional assessment is necessary to determine an individual’s dietary prescription; the methods and tools for this assessment are described in the KDOQI guidelines (3). Additional information about nutritional management in CKD not covered here, including potassium, sodium, and micronutrients, is discussed by KDOQI (3) and will be addressed by the PRNT in future CPRs. As with all documents produced by the PRNT, the practical day-to-day management of the nutritional prescription will be developed by the Taskforce during the dissemination phase of the guideline (4).

## Methods

Existing guidelines on energy and protein requirements for age and gender in healthy children were reviewed and used to help determine recommendations for children with CKD stages 2–5 and when on dialysis (CKD2–5D).

### Terminology

The recommendations for dietary requirements for energy and nutrients, published by national and international health bodies, are expressed in various terms and represent different measures. These are detailed in the Supplementary Tables 1a and 1b.

We have not endorsed any one set of published values for energy and protein requirements but have taken a pragmatic approach and taken the range of requirements from the national health bodies for our recommendations. Since the national and international terms for energy and protein requirements have different definitions (and are therefore not directly comparable), and as we show a range of the published values, we will use a novel term: Suggested Dietary Intake (SDI) for our recommendations. The SDI comprises a range of values. The lower and upper limits of the SDI for energy fall within the average amount given in the published values (i.e., the daily amount of energy considered sufficient to meet the needs of half the population). The lower and upper limits of the SDI for protein fall within the average amount + 2 SD given in the published values (i.e., the daily amount of protein considered sufficient to meet the needs for nearly all (97.5%) of the population) (Supplementary Fig. 1). The SDI may be used for formulating dietary prescriptions and assessing the adequacy of dietary intake in individuals.

### Development process

The full development process for the CPRs and their purpose has been published (4). PICO (Patient, Intervention, Comparator, and Outcome) questions have been developed in order to develop recommendations that provide specific actionable advice, including choosing between alternative approaches in particular clinical situations.

PICO questions

*Population*: Children from birth to 18 years of age with CKD2–5D.

*Intervention*: Nutritional requirements for energy and protein in children at different stages of CKD.

*Comparator*: Nutritional requirements for energy and protein in age-matched healthy children.

*Outcomes*: Energy and protein requirements to support normal growth and development in children with CKD2-5D.

### Literature search

Details of the literature search are described in Supplementary Table 2. After a critical review of the literature for each PICO question, CPRs were derived and graded using the American Academy of Pediatrics grading matrix (5) (Supplementary Table 3). The Delphi method was used as previously described (4) to seek consensus from experts in the field.

## Clinical practice recommendations

## 1. What are the energy requirements for children with CKD stages 2–5D?

1.1 We suggest that the initial prescription for energy intake in children with CKD2–5D should approximate that of healthy children of the same chronological age. (Level B; moderate recommendation).

1.2 To promote optimal growth in those with suboptimal weight gain and linear growth, we suggest that energy intake should be adjusted towards the higher end of the suggested dietary intake (SDI). (Level D; weak recommendation).

1.3 In overweight or obese children, adjust energy intake to achieve appropriate weight gain, without compromising nutrition. (Level X; strong recommendation).

### Evidence and rationale

#### Healthy children

The optimal energy requirement is the energy from food needed to maintain normal body mass, growth, and development, and to support a level of physical activity consistent with long-term good health. Energy requirements are affected by physical activity, growth, lean body mass, genetics, ethnicity, the environment, and current nutritional status.

Recommendations for energy intake come from observational and experimental studies and, more recently, studies using doubly labeled water (DLW) along with a factorial method to address physical activity (6–13). The DLW technique is considered the “gold standard” for assessing total energy expenditure (TEE) in free-living individuals. TEE includes physical activity energy expenditure (the most variable component of TEE), resting energy expenditure (REE), and the thermic effect of food.

There are many guidelines for energy requirements (Supplementary Table 4a) for the healthy population (6–13). The definitions for the terms used are in Supplementary Table 1a.

#### Children with CKD2–5D

The 2009 KDOQI guidelines recommend an energy intake for children with CKD stages 2–5D of 100% of the estimated energy requirement (EER) for chronological age and that further adjustment to energy intake should be based upon the response in terms of the rate of weight gain or loss (3). These statements were level B recommendations. Subsequent studies provide no reason to change these guidelines, but we suggest using the SDI to serve as the standard for energy requirements (Table 1).

**Table 1:** Energy and protein requirements for infants, children and adolescents with CKD2–5D aged 0–18 years

SDI for energy and protein: birth^a^ to 18 years				
Month	SDI^b^ energy (kcal/kg/day)	SDI protein (g/kg/day)	SDI protein (g/day)	
0	93–107	1.52–2.5	8–12	
1	93–120	1.52–1.8	8–12	
2	93–120	1.4–1.52	8–12	
3	82–98	1.4–1.52	8–12	
4	82–98	1.3–1.52	9–13	
5	72–82	1.3–1.52	9–13	
6–9	72–82	1.1–1.3	9–14	
10–11	72–82	1.1–1.3	9–15	
12	72–120	0.9–1.14	11–14	
Year	SDI energy (kcal/kg/day)		SDI protein (g/kg/day)	SDI protein (g/day)
–	Male	Female		
2	81–95^c^	79–92^c^	0.9–1.05	11–15
3	80–82	76–77	0.9–1.05	13–15
4–6	67–93	64–90	0.85–0.95	16–22
7–8	60–77	56–75	0.9–0.95	19–28
9–10	55–69	49–63	0.9–0.95	26–40
11–12	48–63	43–57	0.9–0.95	34–42
13–14	44–63	39–50	0.8–0.9	34–50
15–17	40–55	36–46	0.8–0.9	Male: 52–65 Female: 45–49

For children with poor growth, reference to the SDI for height age may be appropriate. Height age is the age that corresponds to an individual’s height when plotted on the 50th centile on a growth chart

^a^Thirty-seven/40 weeks gestation. Premature infants have higher energy and protein requirements. The increased need for these and other particular nutrients (sodium, potassium, calcium, and phosphorus) must be balanced against the nutritional interventions to control the effects of CKD. This is outside the scope of this CPR

^b^Suggested Dietary Intake (SDI) is based on the Physical Activity Level (PAL) used by the international bodies: 1–3 year PAL 1.4; 4–9 year PAL 1.6; and 10–17 year PAL 1.8. Where guidelines have given a range of energy requirements for different levels of PAL, the lowest PAL has been taken for SDI energy in consideration that children with CKD are likely to have low activity levels

^c^Scientific Advisory Committee on Nutrition (9) reports energy requirements as kcal/day: male 1040 kcal/day; female 932 kcal/day

There have been two strategies to investigate the optimal requirements for energy for children with CKD since publication of the KDOQI guidelines. First, four trials studied REE/basal metabolic rate (BMR) by indirect calorimetry and showed no difference from healthy children after adjustment for lean body mass (14–17). One trial measured BMR by indirect open circuit calorimetry in 20 children with CKD and 20 healthy age- and gender-matched controls (17). The adjusted BMR of children with CKD did not differ significantly from healthy subjects. Second, observational and retrospective studies have reported the effects of specified energy intake measurements in children with CKD (18–40). Most reported that dietary energy intakes of around 100% of EER in children with CKD, managed conservatively or on peritoneal dialysis, resulted in acceptable growth (19–25, 30, 32, 33). The largest prospective trial enrolled 65 children aged 2 to 16 years with an estimated glomerular filtration rate (eGFR) < 75 ml/min/1.73 m^2^ body surface area (BSA) (40). In the 51 completing the study, growth was normal with median energy intakes of 94–98% of estimated average requirement (EAR) at baseline and 85–94% of EAR at 2 years of follow-up.

In children on peritoneal dialysis (PD), energy intake from dialysate must be considered, with reports of 7.5 ± 7 to 9.08 ± 4.13 kcal/kg/day (41, 42), depending on peritoneal glucose exposure (glucose concentration of the dialysate, time on dialysis, cycles, and dwell times) and peritoneal membrane transporter status. While some children may benefit from this additional energy, if there is excessive weight gain, this energy source needs to be taken into account when considering the balance of macronutrients (protein, carbohydrate and fat) provided by the diet.

Indeed, obesity is increasing worldwide, even in children with CKD: the International Pediatric PD Network (IPPN) registry found a 19.7% prevalence of overweight/obesity in children at the start of chronic PD (compared with 8.9% underweight) (2). The Chronic Kidney Disease in Children (CKiD) study has shown the median energy and protein consumption to be higher than recommended in all age groups, implying that at least half the children consumed more energy and protein than recommended (43, 44). Modification of energy intake and lifestyle changes, including physical activity, may be needed (3).

## 2. What are the protein requirements for children with CKD stages 2–5D?

2.1 We suggest that the target protein intake in children with CKD2–5D is at the upper end of the SDI to promote optimal growth. (Level C; moderate recommendation). The protein intake at the lowest end of the range is considered the minimum safe amount and protein intake should not be reduced below this level. (Level X; strong recommendation).

2.2 We suggest that the protein intake in children on dialysis may need to be higher than the SDI for non-dialysis patients to account for dialysate protein losses. (Level C; weak recommendation).

2.3 In children with persistently high blood urea levels, we suggest that protein intake may be adjusted towards the lower end of the SDI, after excluding other causes of high blood urea levels. (Level C; moderate recommendation).

### Evidence and rationale

#### Healthy children

The protein requirement is the amount of protein needed for growth and to balance the losses of nitrogen from the body in individuals maintaining energy balance. We have considered all recent reports citing daily protein needs and we have focused on those using the linear regression approach and the factorial method to inform their guidelines (7, 8, 10, 13, 45–48) (Supplementary Table 4b). Age-appropriate ranges from these guidelines have been adopted to create the SDI for protein. The data from Agence Française de Sécurité Sanitaire des Aliments (AFSSA) (48) have been excluded due to the wide range of values given which lie outside of the ranges of the other guidelines considered.

Protein is a fundamental component of cellular and organ function. Together with sufficient protein provision, there must also be adequate non-protein energy (i.e., carbohydrates, fats) available in order that the carbon skeletons of amino acids are not utilized to meet energy needs (49). Furthermore, unless amino acids are present in the correct balance in an individual’s diet, protein utilization will be negatively affected (49). Dietary protein is needed to maintain protein turnover and to synthesize physiologically important products of amino acid metabolism to lay down as new tissue.

Nitrogen balance can be used to derive estimates of nitrogen protein requirements. The usual approach is based on the regression of nitrogen balance (the equilibrium between intake and loss) on intake. The average protein requirement is based on nitrogen loss by urine, stool, hair, nails, and transpiration (50) plus the extra protein needed for growth (51). The nitrogen amount is calculated and converted to protein with the factor 6.25. The protein requirements for individuals following lacto-ovo vegetarian and vegan diets, in order to achieve the right amount and balance of high quality protein, are 1.2 and 1.3 times higher, respectively, than for people consuming a mixed animal- and plant-based diet (8) due to the lower bioavailability of non-animal protein.

#### Children with CKD2–5D

The 2009 KDOQI guidelines (3) suggested maintaining an intake of dietary protein at 100 to 140% of the dietary reference intake (DRI) for ideal body weight in children with CKD stage 3 and at 100 to 120% of the DRI in children with CKD stages 4 to 5 (level C). In children with CKD stage 5D, it was suggested that dietary protein intake be maintained at 100% of the DRI for ideal body weight plus an allowance for dialytic protein and amino acid losses: for patients on PD 0.15–0.3 g/kg/day and for patients on hemodialysis (HD) 0.1 g/kg/day (level C) (3). Since the publication of the KDOQI guidelines, there have been no studies that support adjustment of the above statements, but we suggest using the SDI to serve as the standard for protein requirements (Table 1).

Two randomized controlled trials (RCTs) have compared low protein versus normal protein diets in children with CKD (52, 53). At 8 months of age, 24 infants with an eGFR < 55 ml/min/1.73 m^2^ were randomized to receive for 10 months either a low protein formula of 1.4 ± 0.3 g/kg/day, with a protein-energy (PE) ratio (the percentage of total dietary energy derived from protein) of 5.6%; or control protein (2.4 ± 0.4 g/kg/day; PE ratio of 10.4%). Significantly lower standard deviation scores (SDS) for length and growth velocity were found in the low protein diet group compared with controls (52). In the second trial, 226 CKD patients aged between 2 and 18 years were randomized to the lowest safe protein intake (0.8 to 1.1 g/kg/day), as recommended by the World Health Organization (WHO) (46), versus an unrestricted protein intake; all patients were advised to have a calorie intake of at least 70% of the WHO recommendations. After 2 years of follow-up, neither the primary endpoint (change in creatinine clearance) nor growth was different between the two groups (53). A Cochrane meta-analysis in 2007 concluded that there was uncertainty over the possibility of harm associated with the provision of strict low protein diets on growth in young infants (54).

Nitrogen balance studies are another option to estimate the protein requirements of a specific population. In a study of 31 children on PD, a dietary protein intake of at least 1.45 g/kg/day (= 144% of recommended daily allowance (RDA)) was required to obtain an estimated nitrogen balance of + 50 mg/kg/day (55).

Based on the available evidence, restriction of dietary protein in the early stages of CKD in children should be avoided. Most importantly, a low protein intake may increase the risk of malnutrition, poor growth, and protein-energy wasting, which are common problems in this population. The ranges for protein SDI given here are a starting point for the initial prescription of dietary protein for feeding a child with CKD. Some children will be consuming a higher protein intake, which can negatively affect acid-base balance and urea levels; phosphate intake is also higher in patients with a high protein intake (56–59). In 20 children on PD, dietary protein intake was negatively correlated with plasma bicarbonate, total body bone mineral density, bone mineral content, and fat-free mass (56). The impact of high phosphate intake is discussed elsewhere (4).

PD is associated with significant protein losses in the dialysate, and losses are higher in small children on chronic PD compared to older children, ranging from 0.28 g/kg/day in infants to 0.1 g/kg/day in adolescents (60); in turn, it is reasonable to suggest that protein intake should be increased accordingly. Peritoneal transport status characteristics and the increase of peritoneal protein losses during peritonitis should also be taken into account. There are no pediatric studies of amino acid and protein losses in extracorporeal dialysis, while amino acid losses as high as 6–10 g/session have been reported in adults treated with HD (61–63).

Children on intensified HD or hemodiafiltration often have an unrestricted diet, as these dialysis modalities allow for better volume and metabolic control (64).

Given the deleterious effects of acidosis and phosphate load in children with CKD, a reduction of protein intake could, in some cases, help improve metabolic control provided that nutritional status is preserved. Urea levels may be used as an indicator of protein intake and may help determine when a reduction in dietary protein intake might be considered. It is not expected that children with CKD2–5D have urea levels in the normal range. Indeed, low levels may indicate insufficient dietary protein. In contrast, urea levels chronically higher than expected for the degree of CKD are most commonly due to excessive dietary protein relative to energy intake but may also be secondary to a catabolic state, acute or chronic dehydration, or steroid therapy. The urea level associated with uremic symptoms is variable with age and between individuals. Chronically high urea levels may be tolerated.

## 3. How is the nutritional prescription for energy and protein provided for children with CKD stages 2–5D?

3.1 Breastfeeding is the preferred method for feeding an infant with CKD. (Level X; strong recommendation).

3.2 When breastfeeding is not possible or expressed breastmilk is not available in adequate amounts for the infant with CKD, we suggest that whey-dominant infant formulas be used. (Level A; strong recommendation).

3.3 We suggest that breastmilk and infant formulas should be fortified when there is a prescribed fluid restriction or when a more energy or nutrient dense feed is required to meet nutritional requirements. (Level A ; strong recommendation).

3.4 We suggest that the concentration of feeds and addition of dietary supplements are prescribed in a gradual manner in order to maximize acceptance and tolerance. (Level D; weak recommendation).

### Evidence and rationale

Breastmilk is the best source of nutrition for infants (65), including those with CKD2–5D who may benefit from its low renal solute load. Standard whey-dominant infant formulas have a protein and electrolyte content closer to that of breastmilk than casein-dominant formulas, so are the preferred alternative and may be beneficial beyond the first year of life. Do not use soya infant formulas under 12 months of age due to their high phytoestrogen content, unless there is a specific medical indication.

The nutritional content of infant formulas may be concentrated in a smaller volume when there is a need for fluid restriction, or when normal feed volumes exacerbate vomiting and gastro-esophageal reflux. Most standard infant formulas are reconstituted to an approximate 13% concentration (i.e., 13 g powder to 100 ml water, providing 67 kcal and 1.3 g protein per 100 ml). It is suggested that this concentration may be increased daily by 1–3%, up to 20% (i.e., 20 g powder to 100 ml water), depending on local practices and the infant’s tolerance of feed changes. Infant formula powder may also be added to expressed breastmilk (EBM) at a concentration of 3–6% (i.e., 3–6 g infant formula powder to 100 ml EBM), increasing the total energy density up to 1 kcal/ml. Breastmilk fortifier may also be used for preterm infants.

Concentrating the formula should be done gradually to ensure tolerance since the increase in osmolality (66) may cause diarrhea, vomiting, and gastro-esophageal reflux. It also increases the renal solute load and may lead to excessive intakes of phosphate, potassium, and other minerals and vitamins. Toxic levels of vitamin A are of particular concern (67). In contrast, the majority of water-soluble vitamins are below the dietary reference intake (DRI) (68, 69). In patients on dialysis, certain vitamins (water-soluble vitamins B1, B5, B6, folic acid, vitamin C) and zinc are lost excessively in the dialysate; these may need to be supplemented over and above the levels provided by the formula to meet recommended intakes. Recommendations regarding the vitamin intake for children with CKD are included in the KDOQI guidelines (3).

An alternative is to use an infant formula at a normal concentration (or EBM if available) and to add protein powder and/or energy modules (glucose polymers, fat emulsions, or combined carbohydrate and fat preparations) to design a patient-specific profile for energy and protein, with attention to vitamin and mineral supplementation as necessary (24, 70, 71). This method of fortification is particularly used if concentrating the infant formula provides an intake of vitamin A, potassium, or phosphate beyond desirable levels. A simple recipe and caregiver education are required to reduce errors in formula preparation (72). Energy and nutrient dense liquid infant formulas are available commercially and may be useful. The addition of glucose polymers, fat emulsions, and protein powders to standard infant formula and EBM can be used for oral feeding or tube feeding. If a mother wishes to continue breastfeeding, this should be prioritized and incorporated into the feeding plan as long as the infant’s overall nutritional intake is not compromised.

To achieve optimal growth in the healthy infant with appropriate deposition of lean and fat tissue, the PE ratio of the infant formula should ideally be within the range 7–12%; a high PE ratio is required for accelerated weight gain or catch-up growth (Supplementary Table 5) (46). In CKD, the challenge is to preserve the PE ratio while adding non-protein energy sources (carbohydrate and fat) to formula in order to control abnormal blood biochemistry, such as elevated urea, phosphate, or potassium levels. In these situations, it is important to ensure that at least the SDI for protein is provided. A PE ratio of 5.3–6.4% supported weight gain and linear growth in children aged 0–2 years, provided at least 100% of protein requirements were given (21), the lower PE ratio reflecting an increase in energy provision rather than a low protein intake.

Glucose polymers may be added in increments, e.g., 1% daily (1 g extra added to 100 ml formula or EBM per day, yielding an additional 4 kcal/100 ml). Gradual increases will allow for identification of the concentration at which the infant becomes intolerant (developing loose stools, increased vomiting). Tolerance to increased carbohydrate concentration depends on the age of the infant, and the maturity and absorptive capacity of the gut, with some infants more tolerant to a more rapid addition of a glucose polymer.

Fat emulsions may be used and should also be added incrementally, e.g., 1% daily (1 ml added to 100 ml formula or EBM per day) to provide an increase of 0.5 g fat per 100 ml (an additional 5 kcal/100 ml). The increased fat content may delay gastric emptying and cause nausea and vomiting. See Table 2 for suggested percentage concentrations of carbohydrate and fat that may be tolerated.

**Table 2:** Suggested addition of energy modules to formulas

Energy module	Age	Amount of CHO/fat module added to formula	Final concentration of CHO/fat in formula (% or g/100 mL)
Glucose polymer	< 6 months	3–5 g (+ 7 g CHO from infant formula^a^)	10–12
	6 months–1 year	5–8 g (+ 7 g CHO from infant formula^a^)	12–15
	> 1 year	8–18 g (+ 12 g CHO from pediatric formula^a^)	20–30
Fat emulsion (50% fat content)	< 1 year	3–5 ml (+ 3.5 g fat from infant formula^a^)	5–6
	> 1 year	9 ml (+ 4.5 g fat from pediatric formula^a^)	9

Adapted from Shaw V (ed) *Clinical Paediatric Dietetics*, 4th edition (2015). Chichester: Wiley Blackwell, page 18

*CHO* carbohydrate

^a^CHO and fat contents of formulas vary

Hyperkalemia occurs as CKD progresses and is more pronounced in patients with metabolic acidosis. Usually reduced potassium intake is indicated (3), but hyperkalemia can result from cell catabolism when there is deficient energy intake. This can be resolved by the addition of energy modules to the infant’s usual formula or EBM.

Protein powders are added to formulas or EBM to provide a specific amount of protein per kilogram of body weight. Additional protein is particularly important for the child on PD to compensate for protein losses in dialysate (60). Protein intake should be increased by at least 0.15–0.3 g/kg/day for children on PD, 0.1 g/kg/day for HD (3). Supplements should be added in small increments, 0.1 g protein/kg/day, and urea levels measured to detect excessive intake (i.e., urea levels above expected for degree of CKD). On occasion, measurement of dialysis protein losses may help guide a plan for protein supplementation.

3.5 Solid foods should be introduced as recommended for healthy infants, with progression to varied textures and content according to the infant’s cues and oral motor skills. We suggest that all children eat a healthy, balanced diet with a wide variety of food choices, as for the general population, taking into account possible dietary limitations. (Level D; weak recommendation).

3.6 Oral feeding is the preferred route whenever possible. Oral stimulation is desirable, even if oral intake is limited, to prevent the development of food aversion. (Level C; weak recommendation).

### Evidence and rationale

Whilst exclusive breastfeeding for the first 6 months of life is recommended by the WHO and supported by a systematic review (65), the age when solid foods are introduced for infants with CKD should be managed individually. Delayed exposure to pureed and more textured foods may cause feeding problems (73). The nutritional content of solid food must be balanced against that provided by the formula in order to achieve optimum intake of energy, protein, and other nutrients. Dietary limitations in potassium and phosphate may be necessary according to the stage of CKD and abnormal blood biochemistry. A low potassium or phosphate formula may be given so that dietary restrictions can be liberalized to allow a greater variety of foods to be offered. A more liberal oral intake may encourage more normal development and behaviors around food.

From 1 year of age, commercially available fortified milk drinks (with a suitably low phosphate and potassium content) may be useful as they contain iron, vitamin D, and n-3 polyunsaturated fatty acids, which may enhance the diet of the toddler with CKD. Also known as “Young Child Formulas,” they are not routinely recommended for healthy children (74).

Infants and young children with CKD2–5D may be reluctant to take an oral diet. At the same time, tube feeding is associated with long-lasting feeding difficulties, such as chewing and swallowing problems, food refusal, panic attacks (75), and poor development of oral motor skills (73). An important aspect of nutritional treatment is therefore to enable as normal development of feeding and eating as possible. When there are significant feeding difficulties, speech and language therapy may help caregivers with the provision of oral feeding and with non-nutritive oral stimulation; input from a psychologist, including family therapy, may be considered (73).

Whilst encouraging an oral diet, there is a need to educate families about healthier food choices at the time of introduction of solids in order to influence later dietary habits. Besides undernutrition, obesity is common in the CKD population and the frequency is similar to that of the normal population. A food frequency questionnaire (FFQ) in 658 children aged 2–18 years with mild to moderate CKD in North America has highlighted some undesirable eating habits (44). Fast foods were a major contributor of sodium, phosphorus, energy, and fat intake, with cow’s milk and milk products contributing 7.7% of energy, 13.8% of protein, 4.6% of sodium, 20.3% of phosphate, and 15.9% of potassium intakes. Dietary and lifestyle changes to achieve weight control are discussed by KDOQI (3).

3.7 We suggest prompt intervention once deterioration in weight centile is noted. Oral nutritional supplementation should be started in children with inadequate dietary intake, after consideration of medical management of correctable causes of reduced intake. (Level B, moderate recommendation).

### Evidence and rationale

Growth is most rapid in the first year of life, and failure to gain weight is the signal for intervention. If weight is static for just 2 weeks in the first 3 months of life, there is a loss of 1 centile; if static for 4 weeks, 2 centiles are lost; at 6 months of age, there is a loss of 1 centile after a 3 week period of no weight gain; and at 9 months, 1 centile is lost after 4 weeks of static weight. Weight and/or head circumference measurements are more sensitive markers of poor growth in infants with CKD when length/height measurements are often inaccurate.

The first step in response to poor weight gain is to address any correctable causes of reduced dietary intake, such as gastro-esophageal reflux/vomiting (by means of feed thickeners, alginates, antacids, histamine H2 receptor antagonists, proton pump inhibitors, sucralfate, prokinetics), acidosis, volume overload, or inadequate dialysis. Children with CKD may also not achieve adequate oral intake due to reduced appetite, altered smell and taste, and abnormal hormone regulation (76–88) (Supplementary Table 6). When inadequate energy and protein intake persists, nutritional supplementation should be instituted. Many studies in children with CKD suggest that growth deteriorates without supplemental feeding and that with dietary intervention by the oral route or with enteral tube feeding, this deterioration can be diminished (18, 21, 22, 29, 36, 38, 40, 43, 89–95). In children with salt-wasting forms of CKD, salt supplementation is also necessary for optimal growth (3).

Energy and protein modules can be added to EBM, standard infant formulas, and infant renal specific formulas. Alternatively, standard pediatric enteral formulas can be fortified if the child will drink them. Standard adult enteral and renal specific formulas can be modified, if necessary, to meet the nutritional requirements of infants and children, but with particular attention to their vitamin and mineral content, which may be excessive.

For those children who are eating, these modules can also be added to normal foods and beverages to meet energy and protein requirements. Where vitamin and mineral intake is also lacking, the first line of treatment is to give nutritionally complete oral liquids (also known as oral nutritional supplements or sip feeds) suitable for toddlers, older children, and adolescents. If there are concerns with elevated electrolyte levels, a palatable high energy pediatric renal specific oral nutritional supplement can be used, when available.

Glucose polymers can be added to beverages, starting with 5% (5 g added to 100 ml) and increasing to 20–30% as needed, and also to “liquid” foods such as porridge/hot breakfast cereals and custards/soft desserts. Sugar and glucose may also be used, but the quantity may be limited due to their sweet taste and osmotic effect on the gut. Extra fat in the form of vegetable margarines or oils (preferably those with a high content of omega-3 fats, such as olive, walnut, or rapeseed oil), and jams, honey, or syrups can be added to foods. Other options include alternative “milks” derived from plants, such as soy, oat, almond, or coconut, without calcium phosphate fortification. It is not advisable to give rice milk to infants and young children due to its high arsenic content. Fruit juices, or alternatively concentrated fruit flavorings (cordials) if there is hyperkalemia, can be provided; protein-free milk replacement drinks, which have low phosphate contents, can serve as a supplement. Glucose absorbed from PD fluid (41, 42) contributes to total energy intake.

It is rarely necessary to add protein modules to foods for children with CKD2–5, but they may be useful for those on dialysis, choosing low phosphate preparations. Most liquid oral renal specific supplements designed for adults are low volume and can be provided between meals in small portions without compromising appetite.

No recommendation can be made for using dietary supplements of essential amino acids and ketoacids in children with CKD. In a trial with 20 children and adolescents, a low protein diet (0.6 g/kg/day) with ketoacid supplements led to a statistically significant (*p* < 0.001) increase in height SD (from − 1.93 ± 1.76 to − 1.37 ± 1.58) when compared to a diet providing the RDA for protein (96). A study of 20 infants with an eGFR < 30–75 ml/min/1.73 m^2^ (not tube fed), and a diet containing 1.8–2.2 g protein/kg/day with supplemental essential amino acids accounting for 20% of the protein intake showed a decrease in weight and height SD from birth to 12 months: − 0.26 to − 1.53 and − 0.56 to −1.63, respectively (97).

3.8 We suggest that supplemental or exclusive enteral tube feeding should be commenced in children who are unable to meet their nutritional requirements orally, in order to improve nutritional status. (Level B, moderate recommendation).

### Evidence and rationale

Several retrospective studies in children with CKD have shown improvement in weight/BMI SD with tube feeding (18, 21, 22, 91–95). All but two of these studies (91, 95) also showed an improvement in height SD. Although most of the available evidence is for children under the age of 2 years, one study reported an increase in weight and height SD specifically for children aged 2–5 years with tube feeding (21). In these studies, tube feeds provided as much as 100% of requirements, while others were supplemental to oral intake.

Three studies have not shown an association between tube feeding and growth. In a questionnaire-based nested case-control study on 137 dialysis patients, where 70% of the children received tube feeds, there were no significant differences in weight or height SDS up to 1 year after dialysis initiation in patients receiving supplemental feedings compared to those not receiving supplemental feedings (38). Two smaller prospective studies also failed to detect an association between energy intake and growth (29, 97).

Commercial formulas and expressed breastmilk are preferred for tube feeding and can be supplemented, if necessary, as described above. Parents wishing to prepare their child’s tube feeds by blending foods should be counseled by a qualified dietitian about safety issues with this practice, including nutritional quality, microbial contamination, and appropriate equipment and administration (98).

When oral or tube feeding is not sufficient or possible, usually as a result of intestinal failure, parenteral nutrition may be needed and the prescription must be tailored for the child with CKD. Intradialytic parenteral nutrition (IDPN) in children is rarely indicated and does not confer a benefit over enteral feeding (99). Studies are scarce (100–102) and no recommendations for its use can be given.

Ongoing oral stimulation is important in tube fed children to help in the transition to normal feeding once they have had a successful transplant (103, 104).

## Results of the Delphi survey

The Delphi survey was sent to 41 pediatric nephrologists and 28 dietitians from 26 countries, with a 68% response rate (27 pediatric nephrologists and 20 dietitians). The names of all respondents are listed under “Acknowledgments” below.

Of the 14 clinical practice recommendation statements, overall, a 94.5% consensus was achieved with a “strongly agree” or “agree” response, 4.1% had a “neutral” response, 1.2% “disagree,” and 0.4% “strongly disagree” response. On analysis of individual statements, 5 received a disagree response, the highest rate being 5.4% to statement 3.1. The respondents queried breastfeeding when there are issues with weight gain and abnormal biochemistry, and commented that supplemental feeding is necessary. These aspects are covered in the rationale supporting statements 3.3 and 3.4. One statement received a strongly disagree response, the highest rate being 5.4% to statement 1.1. The respondents queried whether stage of CKD, the presence of protein-energy wasting and hypercatabolic state should be taken into consideration. The studies supporting this statement included children with CKD2–5D, and most reported that dietary energy intakes of around 100% of that for healthy children resulted in acceptable growth.

The Taskforce team carefully reviewed all of the statements in light of these responses; none required significant change. However, there has been further clarification to the text and tables as suggested by the respondents.

## Summary of recommendations

A summary of recommendations is provided in Table 3.

**Table 3:** Summary of recommendations

	Category	Recommendation	Grade
1	Energy requirements	1.1 We suggest that the initial prescription for energy intake in children with CKD2–5D should approximate that of healthy children of the same chronological age.	Level B; moderate recommendation
		1.2 To promote optimal growth in those with suboptimal weight gain and linear growth, we suggest that energy intake should be adjusted towards the higher end of the suggested dietary intake (SDI).	Level D; weak recommendation
		1.3 In overweight or obese children, adjust energy intake to achieve appropriate weight gain, without compromising nutrition.	Level X; strong recommendation
2	Protein requirements	2.1 We suggest that the target protein intake in children with CKD2–5D is at the upper end of the SDI to promote optimal growth. The protein intake at the lowest end of the range is considered the minimum safe amount and protein intake should not be reduced below this level. 2.2 We suggest that the protein intake in children on dialysis may need to be higher than the SDI for non-dialysis patients to account for dialysate protein losses. 2.3 In children with persistently high blood urea levels, we suggest that protein intake may be adjusted towards the lower end of the SDI, after excluding other causes of high blood urea levels.	Level C; moderate recommendation Level X; strong recommendation Level C; weak recommendation Level C; moderate recommendation
3	Nutritional prescription	3.1 Breastfeeding is the preferred method for feeding an infant with CKD. 3.2 When breastfeeding is not possible or expressed breastmilk is not available in adequate amounts for the infant with CKD, we suggest that whey-dominant infant formulas be used. 3.3 We suggest that breastmilk and infant formulas should be fortified when there is a prescribed fluid restriction or when a more energy or nutrient dense feed is required to meet nutritional requirements 3.4 We suggest that the concentration of feeds and addition of dietary supplements are prescribed in a gradual manner in order to maximize acceptance and tolerance. 3.5 Solid foods should be introduced as recommended for healthy infants, with progression to varied textures and content according to the infant’s cues and oral motor skills. We suggest that all children eat a healthy, balanced diet with a wide variety of food choices, as for the general population, taking into account possible dietary limitations. 3.6 Oral feeding is the preferred route whenever possible. Oral stimulation is desirable, even if oral intake is limited, to prevent the development of food aversion. 3.7 We suggest prompt intervention once deterioration in weight centile is noted. Oral nutritional supplementation should be started in children with inadequate dietary intake, after consideration of medical management of correctable causes of reduced intake. 3.8 We suggest that supplemental or exclusive enteral tube feeding should be commenced in children who are unable to meet their nutritional requirements orally, in order to improve nutritional status.	Level X; strong recommendation Level A; strong recommendation Level A; strong recommendation Level D; weak recommendation Level D; weak recommendation Level C, weak recommendation Level B, moderate recommendation Level B, moderate recommendation

## Research recommendations

We recommend the following areas of study to provide future evidence based recommendations for energy and protein requirements in children with CKD2–5D:

1. To investigate the outcomes (growth, morbidity, mortality, quality of life) associated with the provision of optimum energy and protein by tube feeding after the first 2 years of life
2. To investigate the ideal protein-energy ratio for the optimum deposition of lean mass versus fat mass and growth, assessed with multi-component body composition models
3. To investigate why some children experience growth failure while others show excessive weight gain despite receiving the SDI for energy and protein
4. To investigate oral motor development of infants and young children who are predominantly tube fed to enhance transition to oral feeding

## Electronic supplementary material

ESM 1 (DOCX 384 kb)
