# Supplementary material for: Energy and protein requirements for children with CKD stages 2-5 and on dialysis–clinical practice recommendations from the Pediatric Renal Nutrition Taskforce
Source: Pediatr Nephrol. 2019 Dec 16;35(3):519–31. doi: 10.1007/s00467-019-04426-0 (PMC6968982; doi:10.1007/s00467-019-04426-0)
Supplement: Supplementary file 1 — (DOCX 384 kb) [file 467_2019_4426_MOESM1_ESM.docx]

**Supplementary Material**

Energy and protein requirements for children with

CKD stages 2-5 and on dialysis – clinical practice recommendations

from the Pediatric Renal Nutrition Taskforce

**Terminology**

**For energy**, terms used are: Average Requirement, Daily Energy Requirement, Estimated Average Requirement and Estimated Energy Requirement (Supplementary Table 1a). In summary, the terms represent the daily needs for half the people in a healthy population, or the predicted average, to maintain energy balance with respect to age (or life stage) and gender consistent with good health, assuming individual requirements are normally distributed within a population (Supplementary Figure 1). Half the individuals in a population will need more, and half will need less than the published values.

**For protein** and other nutrients, terms used are: Adequate Intake, Population Reference Intake, Recommended Daily Allowance, Reference Nutrient Intake, Reference Values, Safe Intake, Safe Level (Supplementary Table 1b). In summary, the terms represent the daily amount that is enough to meet the needs for nearly all the people (97.5%) in a healthy population or the average amount + 2 standard deviations (SD), assuming individual requirements are normally distributed within a population. Where there isn’t enough data to calculate an average, the values are the level of protein or nutrient which is assumed to be adequate for the population’s needs with respect to age (or life stage) and gender consistent with good health. If the average intake of an otherwise healthy individual is at or above these values, the risk of deficiency is judged to be very low. If the average intake is below these values, it is likely that some will have an insufficient intake.

**Supplementary Table 1a Definition of terms for energy requirements**

| Average Requirement (AR) | The level of a nutrient in the diet that meets the daily needs of half the people in a typical healthy population. |
| --- | --- |
| Daily Energy Requirement (DER) | The amount of food energy needed to balance energy expenditure in order to maintain body size, body composition and a level of necessary and desirable physical activity, and to allow optimal growth and development of children, deposition of tissues during pregnancy, and secretion of milk during lactation, consistent with long-term good health. For healthy, well-nourished adults, it is equivalent to total energy expenditure. There are additional energy needs to support growth in children and in women during pregnancy, and for milk production during lactation. |
| Estimated Average Requirement (EAR) | An estimate of the average requirement of energy or a nutrient needed by a group of people (i.e. approximately 50% of people will require less, and 50% will require more). |
| Estimated Energy Requirement (EER) | The average dietary energy intake that is predicted to maintain energy balance in a healthy adult of a defined age, gender, weight, height, and level of physical activity consistent with good health. |

**Supplementary Table 1b Definition of terms for protein and nutrient requirements**

| Adequate Intake (AI)* | Is a dietary recommendation used when there isn't enough data to calculate an Average Requirement. An AI is the average nutrient level consumed daily by a typical healthy population which is assumed to be adequate for the population's needs. |
| --- | --- |
| Adequate Intake (AI)* | Established when evidence is insufficient to develop an RDA and is set at a level assumed to ensure nutritional adequacy. |
| Adequacy of nutrient intake (AI)* | Intake of a nutrient that meets the individual's requirement for that nutrient. |
| Population Reference Intake (PRI) | The intake of a nutrient that is likely to meet the needs of almost all healthy people in a population. |
| Recommended Dietary Allowance (RDA)* | Average daily level of intake sufficient to meet the nutrient requirements of nearly all (97%-98%) healthy people. |
| Recommended Daily Allowance (RDA)* | The average daily dietary nutrient intake level sufficient to meet the nutrient requirement of nearly all (97 to 98 percent) healthy individuals in a particular life stage and gender group |
| Reference Nutrient Intake (RNI) | The RNI is the amount of a nutrient that is enough to ensure that the needs of nearly all a group (97.5%) are being met. |
| Reference Values (RV) | RV is the nutrient Intake are the basis on which diets are planned to match nutritional requirements for food intake. In addition, they form the basis for food rules and regulations for the food industry and food monitoring. |
| Safe Intake (SI) | The Safe Intake is used where there is insufficient evidence to set an EAR, RNI or LRNI. The safe intake is the amount judged to be enough for almost everyone, but below a level that could have undesirable effects. |
| Safe Level (SL)* | Adding 1.96 standard deviations (SD) to the average requirement. |
| Safe Level (SL)* | The average requirement of the population group, plus 2 standard deviations. |

**Sources of terminology:** DACH, Deutschland Austria-Confoederatio Helvetica; EFSA, European Food Safety Authority; FAO/WHO/UNU, Food and Agriculture Organization/World Health/Organization/ United Nations University; IOM Institute of Medicine (USA); NHMRC, National Health and Medicine Research Council (Australia and New Zealand); NNR, Nordic Nutrition Recommendations; SACN, Scientific Advisory Committee on Nutrition (UK).

*Publications use different wording in terminology.

**Supplementary Table 2 Search terms strategy used in the literature review for energy and protein requirements**

1980 – August 2019 English language

Medline, PubMed, Embase, Cochrane library, Cinahl, manual searching

Meta-analyses, randomised controlled trials, prospective studies with more than 20 children (no prospective studies in adults)

No retrospective pediatric studies unless a paucity of papers

| 1 | **kidney disease** | **renal failure** | **renal insufficiency** | **chronic kidney disease** | **kidney failure** | **kidney injury** | **kidney dysfunction** | **CKD** |
| --- | --- | --- | --- | --- | --- | --- | --- | --- |
|  | **CRF** | **CKF** | **ESRD** | **ESRF** | **dialysis** | **renal replacement therapy** | **pre dialysis** | **peritoneal dialysis** |
|  | **hemodialysis** | **haemodialysis** | **CAPD** | **APD** |  |  |  |  |
| **2** | **calorie** | **protein** | **energy** |  |  |  |  |  |
| **3** | **diet** | **dietary** | **nutrition** | **food** | **feed** | **intake** | **requirements** | **acceptable macronutrient distribution (AMDR)** |
|  | **dietary management** | **dietary advice** | **dietary restriction** | **supplementation** | **dietitian** | **dietician** |  |  |
| **4** | **enteral nutrition** | **tube feeding** | **gastrostomy** | **oral intake** | **energy intake** | **dietary protein** | **malnutrition** | **malnutrition/prevention and control** |
|  | **nutritional support** | **feeding methods** | **breast feeding** | **human milk** | **infant formula** | **weaning** | **appetite** | **dysmotility** |

**Supplementary Table 3** **American Academy of Pediatrics grading matrix**


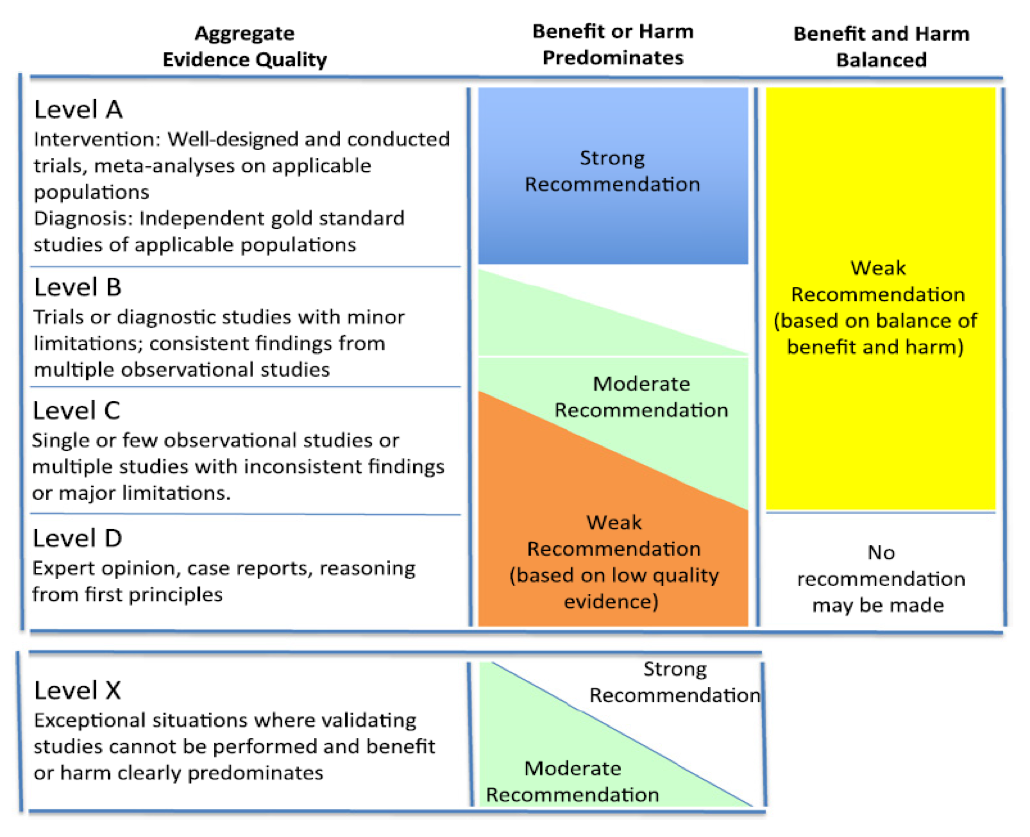


**Supplementary Table 4a**

| **Current recommendations for Energy requirements for healthy children 0-24 months*** | | | | | | | | |
| --- | --- | --- | --- | --- | --- | --- | --- | --- |
|  | **FAO/WHO/**  **UNU**  **2001/2004** | **HCN 2002** | **IOM**  **2005** | **SACN 2011** | **NNR**  **2012** | **EFSA**  **2013** | **DACH**  **2015** | **NHMRC**  **2017** |
|  | DER | EAR | EER | EAR | EAR | AR | AR | EER |
| **Age (month)** | kcal/kg/day | kcal/kg/day | kcal/kg/day | kcal/kg/day | kcal/kg/day | kcal/day | kcal/kg | kcal/kg/day |
| **0** |  | **93/93** | **107/104** |  |  | **nv** | **nv** |  |
| **1** | **113/107** |  | **107/102** | **120/120** | **116/112** |  |  | **109/103** |
| **2** | **104/101** |  | **95/95** |  |  |  |  | **108/102** |
| **3** | **95/94** | **83/83** | **82/83** | **96/96** | **98/97** |  |  | **96/96** |
| **4** | **82/84** |  | **82/82** |  |  |  |  | **86/86** |
| **5** | **81/82** |  | **82/82** | **72/72** |  |  |  | **82/82** |
| **6** | **79/78** | **83/83** | **80/79** |  | **81/82** |  |  | **82/83** |
| **7** | **79/78** |  | **80/79** | **72/72** |  | **644/573** |  | **80/78** |
| **8** | **79/78** |  | **80/80** |  |  | **667/597** |  | **81/80** |
| **9** | **80/79** |  | **82/81** |  |  | **693/644** |  | **80/79** |
| **10** | **80/79** |  | **82/81** |  |  | **716/620** |  | **81/81** |
| **11** | **80/79** |  | **82/81** |  |  | **740/644** |  | **81/81** |
|  |  |  |  |  |  | kcal/kg/day |  |  |
| **12** | **82/80** | **85/83** | **82/81** | **72/72** | **81/80** | **77/76** | **120/118** | **81/81** |
| **15** |  |  | **82/81** |  |  |  |  | **82/81** |
| **18** |  |  | **82/82** |  |  |  |  | **82/83** |
| **21** |  |  | **82/82** |  |  |  |  | **82/83** |

* values given for Male/Female; nv, no value

| **Current recommendations for Energy requirements for healthy children 2-18 years*** | | | | | | | | |
| --- | --- | --- | --- | --- | --- | --- | --- | --- |
|  | **FAO/WHO**  **/UNU**  **2001/2004** | **HCN**  **2002** | **IOM**  **2005** | **SACN 2011** | **NNR**  **2012** | **DACH**  **2015** | **EFSA**  **2017** | **NHMRC**  **2017** |
|  | **DER** | **EAR** | **EER** | **EAR** | **EER** | **AR** | **AR** | **EER** |
| **Age (year)** | **kcal/kg/day** | **kcal/kg/day** | **kcal/kg/day** | **kcal/day** | **kcal/kg/day** | **kcal/kg/day** | **kcal/kg/day** | **kcal/kg/day** |
| **2** | **84/95** | **85/83** | **83/82** | **1004/932** | **81/79** | **95/92** | **81/79** | **83/83** |
|  |  |  |  | **kcal/kg/day** |  |  |  |  |
| **3** | **80/77** |  | **80/76** | **81/77** | **82/80** | **80/76** | **80/77** | **82/77** |
| **4** | **77/74** | **73/66** | **85/82** | **85/81** | **82/79** | **93/90** | **84/82** | **77/73** |
| **5** | **75/72** |  | **79/76** | **80/75** | **77/73** | **84/80** | **80/75** | **71/68** |
| **6** | **73/69** |  | **73/71** | **75/71** | **73/69** | **74/70** | **75/71** | **67/64** |
| **7** | **71/67** |  | **69/67** | **72/67** | **68/65** | **77/75** | **71/67** | **63/60** |
| **8** | **69/64** |  | **65/61** | **67/63** | **66/62** | **69/67** | **66/63** | **60/56** |
| **9** | **67/61** | **63/55** | **62/56** | **63/59** | **63/59** | **61/59** | **63/60** | **57/53** |
| **10** | **65/58** |  | **67/59** | **65/61** | **66/62** | **69/63** | **64/61** | **55/49** |
| **11** | **62/55** |  | **63/55** | **62/57** | **60/56** | **62/56** | **62/57** | **51/45** |
| **12** | **60/52** |  | **59/52** | **59/53** | **58/52** | **55/49** | **59/58** | **48/43** |
| **13** | **58/49** |  | **57/49** | **56/48** | **55/48** | **59/50** | **56/50** | **46/41** |
| **14** | **56/47** | **52/42** | **54/47** | **54/46** | **52/46** | **52/46** | **54/47** | **44/39** |
| **15** | **53/45** |  | **53/45** | **51/45** | **50/44** | **55/46** | **51/46** | **42/38** |
| **16** | **52/44** |  | **51/43** | **49/44** | **49/43** | **51/44** | **50/45** | **40/37** |
| **17** | **50/44** |  | **50/42** | **48/43** | **48/43** | **49/44** | **49/45** | **40/36** |

* values given for Male/Female

**Note:** Energy recommendations include the physical activity level (PAL) used by the international bodies: 1-3 yr PAL 1.4; 4-9 yr PAL 1.6; 10-17 yr PAL 1.8.

**Note:** Reference weights used in compiling recommendations based on body weight are given in Supplementary Table 3c.

**Supplementary Table 4b**

| **Current recommendations for Protein requirements for healthy children 0-12 months*** | | | | | | | | | |
| --- | --- | --- | --- | --- | --- | --- | --- | --- | --- |
|  | **COMA**  **1991** | **HCN**  **2002** | **IOM**  **2005** | **AFSSA**  **2007** | **FAO/**  **WHO/**  **UNU**  **2007** | **EFSA**  **2012**  **accepted WHO/FAO/UNU 2007** | **NNR**  **2012** | **DACH**  **2017** | **NHMRC**  **2017** |
|  | **DRV** | **RDA** | **AI** | **RI** | **SL** | **PRI** | **SL** | **RV** | **AI** |
| **Age (month)** | g/day | g/kg/day  (g/day) | g/kg/day  (g/day) | g/kg/day | g/kg/day  (g/day) | g/kg/day | g/kg/day | g/kg/day  (g/day) | g/day |
| **0** | **12.5** | **1.8**  **(9/8)** | **1.52** | **0.94-2.60** | **Nv** | **nv** | **nv** | **2.5**  **(8)** | **10** |
| **1** |  |  |  |  |  |  |  | **1.8**  **(8)** |  |
| **2** |  |  |  |  |  |  |  | **1.4**  **(8)** |  |
| **3** |  | **1.4**  **(10/9)** |  |  |  |  |  |  |  |
| **4** | **12.7** |  |  |  |  |  |  | **1.3**  **(11)** |  |
| **5** |  |  |  |  |  |  |  |  |  |
| **6** |  | **1.2**  **(10)** |  |  | **1.3**  **(10.2/ 9.4)** | **1.31** | **1.1** |  | **14** |
| **7** | **13.7** |  | **RDA**  **1.2**  **(11)** |  |  |  |  |  |  |
| **8** |  |  |  |  |  |  |  |  |  |
| **9** |  |  |  |  |  |  |  |  |  |
| **10** | **14.9** |  |  |  |  |  |  |  |  |
| 11 |  |  |  |  |  |  |  |  |  |

* values given for Male/Female; nv, no value

**Note:** 0-6 months. Assumed needs based on energy and protein of human breast milk.

| **Current recommendations for Protein requirements for healthy children 1-18 years*** | | | | | | | | | |
| --- | --- | --- | --- | --- | --- | --- | --- | --- | --- |
|  | **COMA 1991** | **HCN**  **2002** | **IOM**  **2005** | **AFSSA**  **2007** | **FAO/**  **WHO/**  **UNU**  **2007** | **EFSA**  **2012**  **accepted WHO/FAO/UNU 2007** | **NNR**  **2012** | **DACH**  **2017** | **NHMRC**  **2017** |
|  | **RNI** | **RDA** | **RDA** | **RI** | **SL** | **PRI** | **SL** | **RV** | **RDA** |
| **Age (year)** | g/day | g/kg/day; (g/day) | g/kg/day  (g/day) | g/kg/day | g/kg/day  (g/day) | g/kg/day | g/kg/day | g/kg/day (g//day) | g/day |
| **1** | **14.5** | **0.9 (14/13)** | **1.05**  **(13)** | **0.94-2.60** | **1.14**  **(11.6/10.8)** | **1.14** | **1.0** | **1.0**  **(14)** | **14** |
| **1.5** |  |  |  |  | **1.03**  **(11.8/11.1)** | **1.03** |  |  |  |
| **2** |  |  |  |  | **0.97**  **(11.9/11.4)** | **0.97** | **0.9** |  |  |
| **3** |  |  |  |  | **0.90**  **(13.1/12.7)** | **0.90** |  |  |  |
| **4** | **19.7** | **0.9 (22/21)** | **0.95**  **(19)** | **0.85-0.90** | **0.87**  **(17.1/16.2)** | **0.86** |  | **0.9**  **(18)** | **20** |
| **5** |  |  |  |  |  | **0.85** |  |  |  |
| **6** |  |  |  |  |  | **0.89** |  |  |  |
| **7** | **28.3** |  |  |  | **0.92**  **(25.9/26.2)** | **0.91** |  | **0.9**  **(26)** |  |
| **8** |  |  |  |  |  | **0.92** |  |  |  |
| **9** |  | **0.9 (36/37)** | **0.95**  **(34)** |  |  | **0.91** |  |  | **40/35** |
| **10** |  |  |  |  |  | **0.91** |  | **0.9 (37/38)** |  |
| **11** | **42.1/41.2** |  |  | **0.78-0.90** | **0.90/0.89**  **(40.5/41.0)** | **0.91/0.90** |  |  |  |
| **12** |  |  |  |  |  | **0.90/0.89** |  |  |  |
| **13** |  |  |  |  |  | **0.90/0.88** |  | **0.9 (50/49)** |  |
| **14** |  | **0.8 (56/49)** | **0.85/0.85**  **(52/46)** |  |  | **0.89/0.87** |  |  | **65/45** |
| **15** | **55.2/45.4** |  |  |  | **0.87/0.84**  **(57.9/47.4)** | **0.88/0.85** |  | **0.9/0.8 (62/48)** |  |
| **16** |  |  |  |  |  | **0.87/0.84** |  |  |  |
| **17** |  |  |  |  |  | **0.86/0.83** |  |  |  |

* values given for Male/Female; nv, no value

**Note:** Reference weights used in compiling recommendations based on body weight are given in Supplementary Table 3c.

**Supplementary Table 4c**

**Reference weights used in international guidelines when determining energy and protein requirements**

The table indicates information regarding reference weights in the source documents used in compiling this clinical practice guideline. Reference weights cited include: median weight for age (WHO, EFSA), 50^th^ centile (SACN), median body mass index and median height for age (IOM), mean of reference values (NNR); those from HCN, DACH, NHMRC are based on growth charts without further specifications.

| **Source** | **Description of Weight Used** |
| --- | --- |
| WHO | **Median weight for age** (infants and children 1–4.99 years) and median weight for height from NCHS/WHO international reference population growth for infants and children (WHO 1983), page 90 [www.fao.org/3/a-y5686e.pdf](http://www.fao.org/3/a-y5686e.pdf) |
| IOM | Calculated from CDC/NCHS Growth Charts (Kuczmarski et al., 2000); **median body mass index and median height for age** 4 through 19 years, page 35 [www.nal.usda.gov/sites/default/files/fnic_uploads/energy_full_report.pdf](http://www.nal.usda.gov/sites/default/files/fnic_uploads/energy_full_report.pdf) |
| HCN | Reference values for weight for age until 18 based on results from the Fourth National Growth Research (Fredriks AM 1997; Frederiks AM 2000; TNO98), page 37 [www.gezondheidsraad.nl/documenten/adviezen/2001/07/18/voedingsnormen-energie-eiwitten-vetten-en-verteerbare-koolhydraten](http://www.gezondheidsraad.nl/documenten/adviezen/2001/07/18/voedingsnormen-energie-eiwitten-vetten-en-verteerbare-koolhydraten) |
| SACN | **50th centile** of UK-WHO growth standards for infants and pre-school children, 50th centile of UK 1990 reference for school-aged children, page 1 <https://assets.publishing.service.gov.uk/government/uploads/system/uploads/attachment_data/file/339317/SACN_Dietary_Reference_Values_for_Energy.pdf> |
| NNR | Values for body weight related to age in the group aged 0–5 years based on the **mean of reference values** from Denmark, Norway, Sweden and Finland. No values were available for 2–5 year olds in Finland so data for 3–5 year olds in Norway and the other Nordic values were used. Values for growth at school age show increasing weight-to-height ratios and an increased prevalence of overweight, therefore, values for 6–17 year olds are based on mean values from 1973–1977, page 172 <http://norden.diva-portal.org/smash/get/diva2:704251/FULLTEXT01.pdf> |
| EFSA | **median weight for age,** page 91 [www.efsa.europa.eu/efsajournal](http://www.efsa.europa.eu/efsajournal) |
| DACH | Based on the German Health Interview and Examination Survey for Children and Adolescents (KiGGS; 2003 – 2006), page 12. <https://www.dge.de/wissenschaft/weitere-publikationen/faqs/energie/#aenderung>  [www.rki.de/EN/Content/Health_Monitoring/Health_Reporting/GBEDownloadsB/KiGGS_referenzperzentile.pdf?__blob=publicationFile](http://www.rki.de/EN/Content/Health_Monitoring/Health_Reporting/GBEDownloadsB/KiGGS_referenzperzentile.pdf?__blob=publicationFile) |
| NHMRC | Reference weight Kuczmarski et al., 2000 (CDC growth charts US),page 16-17 <https://nhmrc.gov.au/sites/default/files/images/nutrient-refererence-dietary-intakes.pdf>; |

**Abbreviations for health bodies**

AFSSA, Agence Française de Sécurité Sanitaire des Aliments; DACH, Deutschland- Austria-Confoederatio Helvetica; EFSA, European Food Safety Authority; FAO/WHO/UNU, Food and Agriculture Organization/World Health Organization/United Nations University; HCN, Health Council of The Netherlands; IOM, Institute of Medicine (USA); NHMRC, National Health and Medical Research Council (Australia and New Zealand); NNR, Nordic Nutrition Recommendations from NCM (Nordic Council of Ministers); SACN, Scientific Advisory Committee on Nutrition (UK); COMA, Committee on Medical Aspects of Food and Nutrition Policy (UK).

**Abbreviations for dietary recommendations used by health bodies**

AI, adequate intake; AR, average requirement; DER, daily energy requirement; DRI, dietary reference intake; DRV, dietary reference value; EAR, estimated average requirement; EER, estimated energy requirement; PRI, population reference intake; RDA, recommended dietary allowance; RI, recommended intake; RNI, reference nutrient intake; RV, recommended value; SL, safe level.

**Supplementary Table 5**

**Energy and protein requirements for accelerated weight gain or catch-up growth for malnourished infants (46)**

| **Rate of weight gain** | **Energy** (kcal/kg/day) | **Protein** (g/kg/day) | **Protein:energy** |
| --- | --- | --- | --- |
| 10g/kg/day | 126 | 2.8 | 8.9% |
| 20g/kg/day | 167 | 4.8 | 11.5% |

Optimal PE ratio for catch-up height is not determined, but is likely to be 11-15%

**Supplementary Table 6**

**Barriers to children with CKD achieving an adequate oral intake.**

| Gastro-esophageal reflux (GER) | A major cause for poor growth in infants: in 22 malnourished infants with CKD (GFR 18.1+-12, range 4-44), feeding/eating behavior was abnormal as assessed by parental questionnaire (76):   - 73% had significant GER - 59% often refused food - 52% vomited excessively - 70% of caretakers were worried about their infant’s nutrition - 78% of carers entertained their child during feeding - 50% bargained with child - 71% force-fed their child |
| --- | --- |
| Dysgeusia | Smell and taste function may be impaired in CKD patients (77):   - Lower mean taste identification scores in paediatric CKD patients compared to controls - Decreasing taste function with decreasing GFR, but no differences in odor identification - No significant association between the total taste identification scores and BMI |
| Appetite-regulating hormones: ghrelin and leptin | Leptin is a hormone produced predominantly by adipose cells.It inhibits hunger.   - Leptin levels elevated in predialysis, HD and PD patients (78,79) - Leptin levels higher in HD patients than in PD patients or controls (80,81), not well eliminated by HD (82) - Leptin levels may be elevated after renal transplant (83) - Inverse correlation between leptin levels and GFR and leptin in some (84), but not all (85) studies - Higher leptin levels in children with a glomerular etiology of CKD compared with children with a non-glomerular cause; higher levels in females than males; higher levels in obese than non-obese children (85)   Ghrelin is a hormone produced in the gastrointestinal tract. Its acylated form induces hunger and increases gastric acid secretion and gastrointestinal motility. Unacylated ghrelin inhibits appetite; increased levels might contribute to protein-energy wasting. Plasma total ghrelin mainly reflects unacylated ghrelin.   - Plasma total ghrelin levels elevated in CKD patients compared to healthy controls and renal transplant patients (83,86) - Negative correlations reported between GFR and total ghrelin levels in plasma (83,87) - Unacylated ghrelin levels higher in CKD patients than controls, highest in HD patients; unacylated ghrelin levels similar in CKD stages I-4, increasing in stages 5 and dialysis (88) - No change in acylated ghrelin levels according to the degree of renal impairment or between CKD patients and healthy controls (83,87,88) - HD eliminates ghrelin to levels comparable to healthy controls after dialysis, whereas ghrelin levels in PD patients are elevated, comparable to conservatively managed patients (82) |

GFR measured in ml/min/1.73 m^2^

**Supplementary Evidence Table 1**

**Energy and protein requirements for children aged 0-18 years with CKD2-5D**

**Table 1a Systematic reviews**

| **Author, Year** | **No. of studies** | **Population, age** | **N** | **Outcomes** | **Meta-analysis model** | **Mean difference of meta-analysis**  **(95% CI)** | **Results** | **Potential bias / limitations** |
| --- | --- | --- | --- | --- | --- | --- | --- | --- |
| Chaturvedi,  2007 (54) | 2 | 250 children | 124 protein restricted diet  126 control diet | Renal deaths (defined as death due to any cause, transplantation or initiation of dialysis)  Creatinine clearance at 2 yr  Weight  Height |  | - RR 1.12, 95% CI 0.54 to 2.33 - MD 1.47, 95% CI -1.19 to 4.14 - MD -0.13, 95% CI -1.10 to 0.84 - MD -1.99, 95% CI -4.84 to 0.86 | - No significant differences in the number of renal deaths. - No difference in GFR changes, growth (height and weight), nutritional status (skinfold thickness, upper arm circumference, serum albumin, serum transferrin), blood pressure, proteinuria, serum lipid level. | Very small number of identified studies |

**Table 1b Randomised controlled trials**

| **Author, Year** | **Population,**  **age** | **N** | **Location** | **Intervention** | **Outcomes reported** | **Potential bias / limitations** |
| --- | --- | --- | --- | --- | --- | --- |
| Uauy, 1994 (52) | Infants  (mean 8 mo)  mean GFR 55 ml/min/1.73 m^2^ | 24 | San Francisco, USA | Average protein intake:  1.4 ±0.3 g/kg/d vs. 2.4 ±0.4 g/kg/d for 10 months    Energy intake 92% RDA for length | - Height at 18 months (low-protein vs. controls): -2.6 ±1.2 versus -1.7 ±0.9 SDS (p<0.05) - Length velocity : -1 SDS vs -0.1 SDS (p<0.05) - Progression of CKD: no progression in both groups | One third of patients received less than the minimum energy intake considered acceptable (80% of RDA for length) on one or more occasions  Short period of follow-up |
| Wingen, 1997 (53) | Children 2-18 yr (mean 10.4 yr)  with GFR 16-60 ml/min/1.73 m^2^ | 191 | Europe | DPI 0.8-1.1 g/kg/day  vs.  no restrictions | - Protein restriction did not affect growth - No effect of diet on the mean decline in creatinine clearance over 2 yr: Progressive group -9.7 ±8 vs -10.7 ±11.8 ml/min/1.73 m^2^ (ns) Non-progressive group -2.5 ± 7.5 vs -4.3± 10 ml/min/1.73 m^2^ (ns) | Significant loss to follow-up, with available data on kidney function dropping from 100% to 88% at follow-up |

**Table 1c Randomised controlled trials – level of evidence**

| **Author, year** | **Sequence generation** | **Allocation concealment** | **Blinding of participants** | **Blinding of personnel** | **Blinding of outcome assessors** | **Incomplete outcome data** | **Selective outcome reporting** | **Other sources of bias** | **Funding source** |
| --- | --- | --- | --- | --- | --- | --- | --- | --- | --- |
|  | Yes/no | Yes/no | Yes/no | Yes/no | Yes/no | Yes/no | Yes/no | Yes/no |  |
| Uauy, 1994 (52) | Unclear | Unclear | Yes | Yes | Yes | Not applicable |  | No | Yes |
| Wingen, 1997 (53) | Yes | Yes | No | No | No | No |  | No | Yes |

**Table 1d Prospective observational studies**

| **Author, Year** | **Patients** | **N** | **Location** | **Intervention** | **Outcomes reported** | **Potential bias / limitations** |
| --- | --- | --- | --- | --- | --- | --- |
| Quan, 1996 (60) | PD patients aged  2 months-18 yr | 25 | Dallas,  USA | Measurement of peritoneal protein losses during continuous cycler PD | - Inverse correlation between daily protein losses and body surface area - Infants had nearly 2-fold greater daily PD protein losses/BSA than older children (average 0.28 g/kg in the first year vs. 0.1 g/kg in adolescents) |  |
| Coleman, 1998 (18) | Chronic dialysis  (18 PD, 4 HD)  median age 2.3 yr (range 0.2-10.3 yr) | 22 | Nottingham, UK | All patients treated with gastrostomy feeding  Follow-up 339 patient months  PD patients:  mean DEI 115% of EAR,  mean DPI 2.5 g/kg/day  HD patients:  mean DEI 122% of EAR,  mean DPI 2.6 g/kg/day | - Mean H SDS: -2.22 at baseline -> -2.06 at end of study (p= 0.005) - Mean W SDS -2.22 at baseline -> -1.16 at end of study (p= 0.001) | No control group |
| Edefonti,  1999 (55) | Children on PD, mean age 11.3 ±4.4 yr | 31 | Milan,  Italy | 42 nitrogen (N) balance studies | - Estimated N balance positive in 36 studies, but >50 mg/kg/day in 21 studies (50%) - A DPI of 1.45 g/kg (=144% RDA) required to obtain an estimated N balance of 50 mg/kg/day in children on PD | N balance was positively correlated with DEI  No multivariate analysis was performed |
| Norman,  2004 (40) | Children aged 2-16 yr with CKD  (GFR <75 ml/min/1.73 m^2^) | 51 | Nottingham, UK | Median EAR were 98%, 98% and 94% for  mild (GFR 51–75 ml/min/1.73 m^2^), moderate (GFR 25–50) and severe (GFR <25) CKD respectively at baseline  and 85%, 94%, and 89% EAR respectively at 2 yr | - Δheight SDS at 2 yr: +0.17, -0.07, +0.10 for mild, moderate, and severe CKD respectively - ΔBMI SDS at 2 yr: -0.11, +0.16, -0.27 for mild, moderate, and severe CKD respectively - A correlation between change in energy intake and change in height SDS was observed in severe CKD (r^2^=0.58, p=0.011). |  |
| Sahpazova, 2006 (19) | CKD children,  mean age 8.85 ±4,13 yr (range 1-16 yr),  GFR 22.5-75 ml/min/1.73 m^2^ | 35 | Skopje, Macedonia | Group 1 (16 pts) - suboptimal DPI (mean DPI 94.79% of WHO recommendations)  Group 2 (19 pts) - adequate DPI (mean DPI 175.45% of WHO recommendations)  In all patients DEI of at least 80’% of WHO recommendations | ΔGFR after 2 years:  - -5.41 ±2.87 vs. -9.53 ±8.61 ml/2 yr (p=NS)  - No difference in anthropometric parameters |  |
| Marques de Aquino, 2008 (16) | Children and adolescents on HD,  mean age 12.3 ±3.1 yr  Healthy controls | 25 pts  25 contr | Sao Paulo, Brazil | Indirect calorimetry | Resting energy expenditure (REE):   - 1067 ±191 vs. 1372 ±290 kcal/day (p<0.01) but when adjusted for lean body mass no difference in REE was observed between groups |  |
| Anderson,  2015 (17) | CKD children,  11.9 ±3.4 yr,  GFR 33.7 ±20.5 ml/min/1.73 m^2^  Healthy controls | 20 pts 20 contr | Southampton, UK | Indirect calorimetry | - Adjusted basal metabolic rate (BMR) of children with CKD did not differ significantly from that of healthy subjects: - 1296 ±318 vs. 1325 ±178 kcal/day (p=0.72) - Within the CKD group, GFR significantly related to BMR (r=0.517, p=0.019) | Inflammation and infection not studied |

**Table 1e Retrospective studies**

| **Author, Year** | **Patients** | **N** | **Location** | **Intervention** | **Outcomes reported** | **Potential bias / limitations** |
| --- | --- | --- | --- | --- | --- | --- |
| Zadik, 1998 (59) | Prepubertal children with ESRD,  mean age 8.7±0.5 yr | 31  (16 HD, 15 PD) | Rehovot, Israel | Growth hormone (GH) treatment in all patients | - Both before the initiation of GH therapy and after the first year of treatment, growth velocity (SDS) was inversely correlated with dietary protein intake and positively correlated with caloric intake |  |
| Van Dyck,  1999 (20) | Infants with GFR <30 ml/min/1.73 m^2^ since birth  Literature controls  (Abitbol, 1993) | 20 | Leuven, Belgium | Prescribed diet:  DEI 110-130% or recommended;  DPI 1.8-2.2 g/kg/day  (no tube feeding) | Height SDS patients vs. controls:  - -1.46 vs. -2.8 at 6 mo  - -1.63 vs. -3.3 at 12 mo  Weight SDS:  - - 1.14 at 6 mo  - - 1.53 at 12 mo | Actual dietary intake not calculated |
| Ledermann,1999 (21) | Children with CKD/ESRD,  mean age 1.6 (range 0-4.9) yr | 35 | London,  UK | Enteral feeding (EF)  0-2-yr group (n=26)  DEI (%EAR): 93.7% at 6 mo pre-EF, 104.2% at start EF, 102.3% at 1 yr, 96.5% at 2 yr  DPI (%RNI): 113.2% at 6 mo pre-EF, 110.2% at start EF, 110.0% at 1 yr, 117.3% at 2 yr  2-5-yr group (n=9)  DEI (%EAR): 81.4% at 6 mo pre-EF, 96.4% at start EF, 85.6% at 1 yr, 88.1% at 2 y  DPI (%RNI): 166.3% at 6 mo pre-EF, 157% at start EF, 172.8% at 1 yr, 172.4% at 2 yr | 0-2-yr group (n=26):   - Weight SDS -3.3 at 6 mo pre-EF, -3.1 at start EF, -1.7 at 1 yr, -1.4 at 2 yr - Height SDS: -2.9 at 6 mo pre-EF, -2.9 at start EF, -2.2 at 1 yr, -2.1 at 2 yr   2-5-yr group (n=9):   - Weight SDS -2.3 at 6 mo pre-EF, -2 at -start EF, -1.1 at 1 yr, -0.9 at 2 yr - Height SDS: -2.8 at 6 mo pre-EF, -2.3 at start EF, -2.0 at 1 yr, -2.0 at 2 yr |  |
| Kari, 2000 (22) | Children with GFR <20 ml/min/1.73 m^2^  median age 0.3 yr (range 0-1.5)  Median follow-up 7.65 yr (1.5-13) | 81 survivi-ng >2 yr (out of 101) | London,  UK | Aim:  DEI 100% EAR for chronological age;  DPI >100% RNI for height age  (81% enteral feeding) | Group 1 (25 pts on conservative treatment):   - Height SDS from -2.34 at 6 mo to -2.26 at 1 yr to -1.93 at 2 yr - BMI from 2^nd^ centile at 6 mo to 9^th^ centile at 1 yr to 25^th^ centile at 2 yr   Group 2 (20 patients treated conservatively, then transplanted (TX)):   - Pre TX height SDS from -1.72 at 6 mo to -1.98 at 1 yr, to -2 at 2 yr - BMI from 9^th^ centile at 6 mo to 9^th^ at 1 yr to 75^th^ at 2 yr   Group 3 (36 PD then TX):   - Pre-TX BMI from 25^th^ centile at 6 mo to 50^th^ at 1 yr to 75^th^ at 2 yr - Pre-TX height SDS in those dialyzed before 6 mo of age from -2.17 at 6 mo to -2.09 at 1 yr to -1.24 at 2 yr. In those started on dialysis after 6 mo of age, height SDS remained stable during conservative treatment and dialysis | Actual dietary intake not calculated |
| Ledermann, 2000 (23) | Infants on PD, median age 0.34 yr (range 0.02-1 yr) | 20 | London,  UK | 18 on Enteral Feeding  Aim: DEI 100% EAR for chronological age;  DPI >100% RNI for height age (2-3 g/kg/day) | - Weight SDS from -1.8 to 0.3 at 1 yr to 0.3 at 2 yr - Height SDS from -1.8 to -1.1 at 1 yr to -0.8 at 2 yr | Actual dietary intake not calculated |
| Parekh, 2001 (24) | Infants <1 yr with polyuric CKD (GFR <65 ml/min/1.73 m^2^)  Controls  Historic controls (n=42)  Literature controls (n=12) | 24 | USA | Enteral formula diluted with water to a caloric density of 0.3-0.5 kcal/ml and NaCl 2-4 mEq/100 ml of formula. Average DEI 104 kg/kg/day (102% RDA), average DPI 2.45 g/kg/day (153% RDA) | Multivariate analysis:   - ∆Height SDS at 1 yr +1.37 in the treatment group vs. historic controls (p=0.017) - ∆Height SDS at 2r y +1.83 in the treatment group vs. literature controls (p=0.003) |  |
| Azocar,  2004 (56) | Children on PD,  mean age 5.84 yr (range 0.16-14.66 yr) | 20 | Santiago de Chile, Chile | DEXA, anthropometry, dietary intake, biochemistry | - At months 1 and 6, the DPI was 144.3% and 129.9% respectively, and showed a negative correlation with bone mineral density, bone mineral content and fat free mass (p<0.05). - DPI negatively correlated with plasma bicarbonate at month 1 (p<0.05) |  |
| Laakkonen, 2008 (25) | Children <2 yr treated with continuous PD  Mean age at onset of PD: 0.4 yr | 23 | Helsinki,  Finland | Mean DEI 110-120% of RDA, mean DPI 2-3 g/kg/day (mostly through NGT) | - In pts who had been on PD for at least 9 mo (n=16), mean height SDS at onset was  -1.9±1.2 and -1.6± 1.8 after 9 months  - Catch-up growth in 64% of pts | Actual dietary intake not calculated |

**Abbreviations**

BMI body mass index; BMR basal metabolic rate; BSA body surface area; CKD chronic kidney disease; DEI daily energy intake; DPI daily protein intake; EAR estimated average requirements; EF enteral feeding; ESRD end-stage renal disease; GFR glomerular filtration rate; GH growth hormone; HD hemodialysis; NGT nasogastric tube; NS not significant; PD peritoneal dialysis; RDA recommended daily allowance; RNI reference nutrient intake; REE resting energy expenditure; SDS standard deviation score; TX transplant.

**Supplementary Evidence Table 2**

**Diet prescription for children aged 0-18 years with CKD 2-5D**

**Table 2a Dietetic input - prospective studies**

| **Author, Year** | **Population, age** | **N** | **Location** | **Intervention** | **Results** | **Potential bias / limitations** |
| --- | --- | --- | --- | --- | --- | --- |
| Arnold, 1983 (89) | 2.5–11 yr  CKD with growth failure | 12 | USA | Non-randomised trial  1^st^ year nutritional counselling. 8/12 received less than 75% RDA energy.  2^nd^ year nutritional counselling and caloric supplement (glucose polymer) to receive 100% RDA energy. | - Energy intake increased from 73% +- 5 to 103% +-6 of RDA (p<0.01) - Growth rate increased from 3.9+-0.58 to 5.68+-0.26 (p<0.05) - Height SD -2.95 to -3.74 in unsupplemented period, -3.46 to -3.69 in supplemented period - Increase in skinfold thickness, MUAC, S-Alb, P-Chol, P-Tg - Children who ingesting >75% RDA prior to starting supplementation did not increase their growth rates as did the children ingesting <75% RDA | No control group |
| Coleman, 1999 (90) | CPD at a mean age of 7.7 yr (range, 0.2 to 8.5 yr) | 13 | UK | Quantitative data regarding dietetic contacts and growth parameters  8 children received tube feeding (7 gastrostomy, 1 NG) in combination with CPD at a mean age of 4.3 years (range, 0.2 to 8.2 yr).  All children under 5 yr of age tube fed. | - 781 dietetic contacts during 182 patient months of observation, mean of 5.9 contacts per patient, per month in children <5 yr of age, compared with 3.1 (SD 1.6) contacts in children >5 yr of age - 82% of contacts were with children receiving nutritional support via a button - Telephone contact accounted for 41% of all contacts in children <5 yr of age, compared with 7% in children > 5 yr of age - Mean SDS for height and weight at the start were -1.2 and -1.32, and at the end were -1.14 and -0. 73 - BMI SDS improved from -0.91 to 0. 17 (p = 0.03) | No control group |

**Table 2b Enteral tube feeding – prospective studies**

| **Author, Year** | **Population, age** | **N** | **Location** | **Intervention** | **Outcomes reported** | **Potential bias / limitations** |
| --- | --- | --- | --- | --- | --- | --- |
| Abitbol, 1993 (29) | Renal insufficiency diagnosed within 1 mo of birth, followed until 2 yr of age.  GFR 6-38 ml/min/1.73 m^2^ at 3 mo -> 5-69 at 2 yr. | 12 | USA | Energy 95% (range 63-150%) RDA, protein 141% (range 94-205%) RDA.  Concentrated formula 81 kcal/100ml.  3 NGT  3 gastrostomy  6 orally | - No association between nutrient or energy intake and growth - No association between degree of renal insufficiency and growth - Growth stabilised at -2 SD, no catch-up growth | No control group |
| Ellis, 2001 (38) | Children with CKD;  64 non-survivors matched with 110 survivors aged 0-5 yr.  126 PD, 8 HD, 3 unknown. | 137 | USA | Nested case-control study  Supplemental tube feed given to 70% of the patients.  Started within 6 mo from initiation of dialysis. | - Questionnaire returned for 51 non-survivors and 86 survivors - Supplemental tube feeding more common in under 2 yr olds (approx. 80% vs. 41%), patients with comorbidities (84% vs. 16%), patients with GER (95% vs 61%), p<0.001 - No differences with enteral feeding in weight or height SDS at 30 days, 6 months, and 1 year after dialysis initiation in those patients receiving supplemental feedings compared to those not receiving supplemental feeding, nor in the change in weight or height SDS from 30 days to 6 months or 1 year after dialysis initiation - NG tubes more common in under 2 yr olds and gastrostomy in 2-5 yr olds. - No association with mortality | Based on a questionnaire  No information on formula type, or energy and protein intake  No information on oral supplements |
| Norman, 2004 (40) | 35 controls, GFR >75 ml/min/1.73 m^2^; mean GFR 104 ml/min/1.73 m^2^ (SD 18.9) (follow-up for HUS);  23 mild CKD, GFR 50–75;  19 moderate CKD, GFR 25–50.  Age range 2-16 yr. | 51 | UK | Follow-up for 2 yr.  Glucose polymer in 5% of mild, 10% of moderate, 38% of severe CKD.  Complete enteral feed in 8% of patients. | - All children who were taking energy supplements consistently and completed the 2 years reported energy intakes that exceeded 80% EAR and exhibited an increase in height and/or BMI SDS - A correlation between change in energy intake and change in height SDS was observed in severe CKD (r2=0.58, p=0.011). |  |
| Van Dyck, 1998 (91) | Children with CKD, mean GFR 23 ml/min/1.73 m^2^, <35 in all.  Age up to 3 yr. | 15 | Holland | Follow-up 3 yr.  Treatment: protein-restricted, energy-enriched diet, with supplements of sodium chloride, sodium bicarbonate, calcium and vitamin D, but no tube feeding or growth hormone. | Growth at:   - birth; 3mo; 12 mo; 24 mo; 36 mo - Head circumference SDS -1.02; -0.82; -0.75; -0.33 - Height SDS -0.45; -1.68; -1.68; -1.48; -1.96 - Weight SDS -0.27; -1.04; -1.54; -1.48; -1.37 |  |

**Table 2c Enteral tube feeding – retrospective studies**

| **Author, Year** | **Population, age** | **N** | **Location** | **Intervention** | **Results** | **Potential bias / limitations** |
| --- | --- | --- | --- | --- | --- | --- |
| Balfe, 1990 (92) | PD patients  Age 3.9+-3.8 yr, (range 0.3-12.8 yr at the beginning). | 20 | Canada | Patients tube fed for 14.1+-11.4 mo, range 1.4-43.2 mo.  4 Nissen fundoplication, 5 PEG, 13 surgical gastostomies.  Dietary prescription: Low-phosphorus formula + concentrated formula + glucose polymer + corn oil + protein supplement if necessary. | Improvement in weight gain (p=0.032) but not on linear growth:   - Before enteral feeds - Wt SDS -2.312 (1.07) to -1.58 (1.09) - Ht SDS -3.18 (1.1) to -3.25 (0.88) - After enteral feeds - Wt SDS -2.38 (0.970 to -1.82 (1.34) - HtSDS -3.28 (0.94) to -3.23 (0.89) | No control group |
| Coleman, 1998 (18) | 18 PD, 2 HD,  2 PD to HD.  Median age 2.3 yr (range 0.2-10.3 yr). | 22 | UK | Follow-up for 339 patient-months.  Feeds: to obtain 100% of EAR for energy. In infants, 115-150 kcal/kg and 2-3 g/kg prot.  Overnight feeds for all, daytime boluses if necessary + supplements (glucose polymer + fat + protein supplement) as needed. Formula low in P and K for 2 infants. For older children, renal-specific complete supplements and energy supplements. 9 patients with oral glucose polymer during the day. | - Height increased from -2.22+-0.4SD at the start to -2.06+-0.37 SD at the end (p=0.005) - Weight SD increased from -2.22+-0.37 to 1.16+-0.34 (p=0.001) - Energy intake in PD patients (not incl. energy from dialysate) 115%+-10.3SD (range 98-131%) of EAR, 105 +-17.8SD kcal (range 72-128 kcal/kg) for <1yr. Mean energy intake 128 kcal/kg - Mean protein intake 2.5+-0.5SD /kg/day (range 1.7-3.4g/kg) - In HD patients 112%+-22SD (range 107-155%) of EAR, 99+-21 kcal/kg (range 84-129 kcal/kg), prot 2.6+-0.6g/kg (range 1.9-3.3 g/kg) - Mean total energy from feeds 61%+-19.7 (range 33-95%) and prot 61%+-23.6 (range 23-98%) | No control group |
| Kari, 2000 (22) | CKD before 6 mo with GFR<20 ml/min/1.73 m^2^ by 1-2 yr of age, or dialysed or RTx by 2 yr of age.  Median age 0.3 yr, range 0-1.5 yr.  101 –> 81 survivors.  25 conservatively managed,  20 pre-emptive RTx,  36 dialysed until RTx. | 101 | UK | Follow-up 7.6 (1.5-13) yr.  Enteral feeding from 0.7 (1-4.5) yr, duration 1.9 (0.1-6.8) yr.  46% with gastrostomies, 22% with Nissen’s fundoplication.  Feeds to provide 100% EAR for energy (chronological age) and 100% RNI for protein (height age).  Whey-dominant infant formula for <2yr old and whole-protein enteral feed + fat/carbohydrate supplements; assessed and adjusted at each outpatient visit (~monthly).  For <1yr, 50% of feed overnight continuous plus daytime boluses.  Frequent small feeds or continuous if vomiting; Nissen’s fundoplication if necessary. | Mean (SD) height -2.16 (1.34) at 6 months (*N* = 63) increased to:   - -1.97 (1.37) at 1 yr (*N* = 75), - -1.79 (1.29) at 2 yr (*N* = 75), - -1.33 (1.29) at 3 yr (*N* = 68, *p =* 0.0006), - -1.27 (1.04) at 5 yr (*N* = 47, *p* = 0.0001), - -0.85 (0.82) at 10 yr (*N* = 18, *p* = 0.001).   Enteral feeding stopped in 97.5% by 6 mo post Tx.   - Height SD increased with tube feeding. - No worsening of uremic hyperlipidemia. | No control group  Energy and protein intakes not reported |
| Ledermann, 1999 (21) | CKD: 29 conservatively managed, GFR 12.1 ml/min/1.73 m^2^ (6–26).  6 PD.  Mean age at the start 1.6 (0–4.9) yr.  26 children under 2 yr, 9 children 2-5 yr. | 35 | UK | Mean duration of enteral nutrition 30.8 (range 12–60) mo.  20 on NGT (6 converted to gastrostomy + Nissen fundoplication), 1 PEG, 1 gastrostomy + Nissen fundoplication.  Continuous overnight feeds + daytime boluses if necessary to provide at least 100% of EAR for energy for chronological age and at least 100% RNI protein for height age.  Whey-dominant/low P+K feeds for <2yr and whole protein feed with energy supplements (glucose polymer, fat emulsion) for >2yr. | For < 2 yr olds:   - Weight SDS –3.1 (1.3) at the start of enteral feeds, increased to –1.7 (1.4) (*P*=0.0003) at 1 yr and to -1.4 (1.8) (*P*=0.0008) at 2 yr - Height SDS –2.9 (1.2) at the start of enteral feeds, increased to –2.2 (1.2) (*P*=0.008) at 1 yr and –2.1 (1.3) (*P*=0.004) at 2 yr - Energy 104.2% (26.2) of EAR at start, 96.5% (16.3) at 2 yr - Non-protein energy increased from 103.1 to 129 kcal/100ml from start to 2 yr - Approximately 80% of energy derived from feeds - Carbohydrate concentration tolerance increased with age - Protein intake 110-117% of RNI   For 2-5 yr olds:   - Weight SDS –2.0 (1.1) at the start of enteral feeds, increased to –1.1 (1.3) (*P*=0.002) at 1 yr and to -0.9 (1.0) (*P*=0.04) at 2 yr - Height SDS –2.3 (0.7) at the start of enteral feeds, increased to –2.0 (0.7) at 1 yr and –2.0 (0.8) at 2 yr - Energy 96.4% (14.9) of EAR at start, 88.1% (18.5) at 2 yr - Approx. 60% of energy derived from feeds - Non-protein energy increased from 108.6 to 153 kcal/100ml from start to 2 yr - Carbohydrate concentration tolerance increased with age - Protein intake 157-172% of RNI   P:E ratio in feeds: start 1 yr 2 yr   - 0-2 yr olds: 6.4 (1.8) 5.3 (1.4) 5.6 (1.5) - 2-5 yr olds: 6.6 (1.2) 7.9 (2.4) 7.4 (2.9) | No control group |
| Mekahli, 2010 (93) | GFR <20 ml/min/1.73 m^2^  Age 0.3  (0-1.5) yr | 101 | UK | Age at start of enteral feeds 0.8 yr (range 0-4.9).  At stop 2.5 yr (range 0.1-8.7).  Median follow-up 13.90 yr (range 0.03 -22.90).  66% tube fed,  37% with gastrostomy, 13% with Nissen fundoplication. | Growth, height SDS (SD):   - -0.42 (2.34) at birth (*n* =40) - -2.07 (1.34) at 0.5 yr (*n=57*) - -1.93 (1.38) at 1 yr (*n=*72) - -1.14 (1.14) at 5 yr (*n=*67) - -1.04 (1.15) at 10 yr (*n=*62) - -1.84 (1.32) at 15 yr (*n=*40) - -1.68 (1.52) at >=18 yr of age (*n=*32) - Tube feeding was associated with catch-up growth in children with and without comorbidity, although better growth was seen in the otherwise normal children - Normal BMI in all (no induction of obesity with tube feeding) - Catch-up growth with tube-feeding also in children over 2 yr of age | No information on diet |
| Rees, 1989 (94) | 38 patients with CKD.  Age range 0.2-9.1 yr. | 38 | UK | 10/16 <2 yr of age tube fed. Tube feeding not reported for >2 yr of age. | - In under 2 yr olds, 60% of tube fed improved growth; 33% improved without tube feeding. | No information on diet |
| Rees, 2011  Registry study (95) | International Pediatric Peritoneal Dialysis Network registry.  Children on chronic peritoneal dialysis  age <2 yr at the beginning of dialysis | 153 | 69 centers in 25 countries around the world | 57 fed on demand, 54 by NGT, 10 by gastrostomy; 52 continuous NGT feeding, 6 discontinued NGT feeding, 26 switched to gastrostomy.  BMI SDS and height SDS were similar initially. | - BMI SDS and height SDS decreased in non-enterally fed - Median (IQR) change in BMI SD -0.41(1.91) SDS/year during on demand feeding vs. +0.97(3.43) SDS/year during NG tube feeding (p< 0.0005) and +1.24 (3.24) SDS/year during gastrostomy feeding (p <0.05) - Height -1.35 (2.63) SDS/year during on demand and -0.72 (1.59) SDS/year during NG tube feeding, and -0.50 (2.47) SDS/year during gastrostomy feeding (p<0.05 for gastrostomy vs. demand feeds) | No information on diet |
| Sienna, 2010 (96) | CKD patients with GFR 13.8 ml/min/1.73 m^2^ (3.9-61.8)  Age 1.7 yr (0.9-15.60 yr) | 102 | Canada | 20 tube fed  82 demand fed  Duration 2.9 (0.9-11.8) yr | - Mean (SD) BMI-for-age –1.22±1.68 at start, 0.43±0.86 at removal, 0.68±1.23 5 yr later - Mean (SD) HtSDS-for-age –2.35±1.86 at start, –1.51±0.99at removal, –1.58±1.64 5 yr later - Mean Wt SDS-for-age –2.53±1.85 at start, –0.66±0.97 at removal, –0.16±0.84 5 yr later   Controls over 5 preceding yr:   - Mean (SD) BMI-for-age 0.30±1.47 and 0.23±2.62 - Mean (SD) HtSDS-for-age –1.04±1.38 and –1.17±1.16 - Mean Wt SDS-for-age –0.33±1.48 and –0.30±1.97 - There was a significant difference in Wt- and BMI-for-age z scores among subjects (p<0.001 and p<0.02, respectively) over the entire study period (from g-tube insertion to 5 y post-removal), but not for Ht-for-age (p=0.642) - Approximately 36% of the non-tube-fed comparison population and 50% of the tube-fed subjects were overweight or obese, but this was associated with steroid post-transplant |  |

**Table 2d Enteral tube feeding – cross-sectional studies**

| **Author, Year** | **Population, age** | **N** | **Location** | **Intervention** | **Results** | **Potential bias / limitations** |
| --- | --- | --- | --- | --- | --- | --- |
| Hui, 2017 (43) | CkiD study.  CKD stages 1-4.  Median age IQR 11 yr (8–15 yr). | 658 | USA | FFQ | - 4% children used supplemental feeds | Type of supplemental feed and association with growth not reported |
| Norman 2000 (36) | ‘Normal’: GFR >75 ml/min/1.73 m^2^ [104 (18.9)] n=35.  Mild CRI: GFR 50–75 n=23.  Moderate CRI: GFR 25–50 n=19.  Severe CRI: GFR <25 n=18.  Age range 2-16.9 yr. | 95 | UK | Dietetic review.  Dietetic therapy at the beginning: glucose polymer for 4% of mild CRI, 11% of moderate and 28% of severe CRI. Complete enteral nutrition: 6% in severe CRI.  Milk substitute in 17% in severe CRI. | - Habitual energy intake lower in severe CRI than in GFR >75, but the use of nutritional supplements brought the suboptimal energy intakes from 85% (SD 27) to 96% (SD 22) (p = 0.04) more similar to the ‘normal’ and mild group, for children with both moderate and severe CRI. - Energy intake prior to supplementation correlated positively with GFR. | No knowledge about the diet before initiation of energy supplements – possibility of reverse causation for habitual energy intake |

**Table 2e Diet modifications**

| **Author, year** | **Population, age** | **N** | **Location** | **Intervention** | **Results** | **Potential bias/limitations** |
| --- | --- | --- | --- | --- | --- | --- |
| Chen, 2017  Cross-sectional study (44) | CKiD cohort  median (IQR) GFR 53.5 ml/min/1.73 m^2^ (38.9-73.5)  age 2-18 yr  Median (IQR) Weight z-score 0.1 (-0.8, 1.0)  Height z-score −0.5 (−1.3, 0.3)  BMI z-score 0.5 (-0.3, 1.4)  % with hypoalbuminemia 7.8,  hyperkalemia 7.0,  hyperphosphatemia 13.1 | n = 658 | USA and Canada | Food frequency questionnaire (FFQ) | In 2-3 yr olds:   - Energy 101 (85, 137)kcal/kg/day - Protein 3.4 (3.0, 4.8) g/kg/day (13 E% (12, 15))   In 4-yr olds:   - Energy 86 (63, 109) kcal/kg/day - Protein 2.8 (2.1, 3.8) g/kg/day (14 E% (12, 15))   In 9-13 yr olds:   - Energy 46 (32, 65) kcal/kg/day - Protein 1.6 (1.1, 2.2) g/kg/day (14 E% (12, 16))   In 14-18 yr olds:   - Energy 37 (27, 51) kcal/kg/day - Protein 1.3 (1.0, 2.0) g/kg/day (14 E% (12, 15)) - No differences in energy or food intake between eGFR <60 or >60 - Milk contributes 7.7% of energy, 13.8% of protein, 4.6% of sodium, 15.9% of potassium (all age groups) | Cross-sectional study  Did not report food intakes in age groups |
| Ellis, 1995  Retrospective (70) | Infants on dialysis (CAPD/CCPD, 12 initially on HD/HDF).  Age at initiation 56 days (range 3-336 days) | 21 | USA | Average follow-up time on dialysis 10 mo (range 1-41 mo).  NGT feeds in 20/21.  Renal specific formula in 14 infants.  MCT oil, protein, glucose polymer, soy formulas or hydrolysed formulas as needed. | - Energy intake 453 +-92 kJ/kg (108 +-22 kcal/kg) | Growth not properly reported  No control group  Descriptive report |
| Parekh, 2001  Prospective follow-up (24) | 24 cases with polyuric chronic renal insufficiency, diagnosed before 1 yr, creatinine  clearance <65 ml/min/1.73 m2  + 54 historic population controls and literature controls | 24 | USA | Nutritional support: enteral formula diluted with water to a caloric density of 0.3 to 0.5  kcal/ml and supplemented with 2 to 4 mEq of sodium per 100 ml of formula.  Treatment group: 104 kcal/kg per day (102% RDA), protein 2.45g/kg per day (153% RDA).  Literary control group: 87% of the RDA for energy and 141+- 42% of the RDA for protein intake.  18/24 received tube feeding.  Renal specific formula with supplements was used until age 2 yr. | - At 1 and 2 yr of age height ΔSDS by regression analysis, adjusted for creatinine clearance, was significantly greater in the treatment group vs. the literature control (+1.37, p = 0.017 and +1.83, p = 0.003 at 1 and 2 yr, respectively) | No real control groups  Better growth could be attributed to better e  Age not reported |

**Abbreviations**

BMI body mass index; CAPD continuous ambulatory peritoneal dialysis; CCPD continuous cycling peritoneal dialysis; CKD chronic kidney disease; CRI chronic renal insufficiency; EAR estimated average requirements; GER gastro-esophageal reflux; GFR glomerular filtration rate; HD hemodialysis; HUS hemolytic uremic syndrome; IQR interquartile range; MUAC mid-upper arm circumference; MCT medium chain triglycerides; NGT nasogastric tube; P:E protein to energy ratio; PD peritoneal dialysis; PEG percutaneous endoscopic gastrostomy; RDA recommended daily allowance; RNI reference nutrient intake; SD(S) standard deviation (score); Tx transplant.
